# Contribution of structural and functional MRI in predicting response to motor training in multiple sclerosis

**DOI:** 10.1177/13524585251398386

**Published:** 2025-12-19

**Authors:** Tetsu Morozumi, Paolo Preziosa, Alessandro Meani, Elisabetta Pagani, Paola Valsasina, Chiara Arezzo, Francesco Romanò, Matteo Albergoni, Massimo Filippi, Maria A Rocca

**Affiliations:** Neuroimaging Research Unit, Division of Neuroscience, IRCCS San Raffaele Scientific Institute, Milan, Italy; Vita-Salute San Raffaele University, Milan, Italy; Neuroimaging Research Unit, Division of Neuroscience, IRCCS San Raffaele Scientific Institute, Milan, Italy; Vita-Salute San Raffaele University, Milan, Italy; Neurology Unit, IRCCS San Raffaele Scientific Institute, Milan, Italy; Neuroimaging Research Unit, Division of Neuroscience, IRCCS San Raffaele Scientific Institute, Milan, Italy; Neuroimaging Research Unit, Division of Neuroscience, IRCCS San Raffaele Scientific Institute, Milan, Italy; Neuroimaging Research Unit, Division of Neuroscience, IRCCS San Raffaele Scientific Institute, Milan, Italy; Neuroimaging Research Unit, Division of Neuroscience, IRCCS San Raffaele Scientific Institute, Milan, Italy; Neuroimaging Research Unit, Division of Neuroscience, IRCCS San Raffaele Scientific Institute, Milan, Italy; Neuroimaging Research Unit, Division of Neuroscience, IRCCS San Raffaele Scientific Institute, Milan, Italy; Neuroimaging Research Unit, Division of Neuroscience, IRCCS San Raffaele Scientific Institute, Milan, Italy; Vita-Salute San Raffaele University, Milan, Italy; Neurology Unit, IRCCS San Raffaele Scientific Institute, Milan, Italy; Neurorehabilitation Unit, IRCCS San Raffaele Scientific Institute, Milan, Italy; Neurophysiology Service, IRCCS San Raffaele Scientific Institute, Milan, Italy; Neuroimaging Research Unit, Division of Neuroscience, IRCCS San Raffaele Scientific Institute, Milan, Italy; Vita-Salute San Raffaele University, Milan, Italy; Neurology Unit, IRCCS San Raffaele Scientific Institute, Milan, Italy

**Keywords:** Multiple sclerosis, MRI, rehabilitation, aerobic training, motor function

## Abstract

**Background:**

Aerobic training is a promising rehabilitation strategy in multiple sclerosis (MS). However, individual responses to exercise vary.

**Objectives:**

To assess whether baseline demographic, clinical, and MRI features in MS patients predict response to aerobic or non-aerobic training.

**Methods:**

Eighty-eight MS patients were randomized to 24-session aerobic (*n* = 43) or non-aerobic (*n* = 45) training over 2–3 months. Responders were defined by a ⩾ 21.6 meters improvement in the 6-minute walking test. Baseline assessments included global disability, fatigue, walking speed, peak oxygen consumption, and structural/functional MRI (lesion load, brain volumetrics, cortical thickness, white matter (WM) microstructural integrity, and resting-state functional connectivity). Random forest models identified baseline demographic, clinical, and MRI predictors of training response.

**Results:**

Thirty-four MS patients (aerobic = 20, non-aerobic = 14) were responders. Predictors of treatment response were corticospinal tract (CST) and middle cerebellar peduncle (MCP) fractional anisotropy in the aerobic group and superior cerebellar peduncle (SCP) mean diffusivity in the non-aerobic group. SCP, MCP, and CST diffusivity metrics predicted training response in whole MS cohort. Repeated cross-validation confirmed identified predictors relevance (median area under the curve from 0.648 to 0.672).

**Conclusion:**

Microstructural integrity of motor-related WM tracts predicts training response in MS, highlighting the role of neuroimaging in identifying patients likely to benefit from rehabilitation.

## Introduction

Multiple sclerosis (MS) is a chronic inflammatory, demyelinating, and neurodegenerative disease of the central nervous system associated with various clinical manifestations.^1^ Therapeutic strategies focus on reducing inflammatory activity, slowing disease progression, and managing symptoms.^1^ Among these strategies, physical rehabilitation is pivotal in enhancing patients’ functional independence and quality of life.^2^

Aerobic training has gained increasing attention as a therapeutic approach for MS.^3^ Beyond improving cardiorespiratory fitness, it has the potential to positively influence brain health by promoting neuroplasticity.^4^ However, individual responses to aerobic training and physical training exhibit considerable variability. Factors contributing to heterogeneity include training modalities, intensity, frequency, and duration, as well as patients’ baseline characteristics. For instance, younger age and lower baseline cardiorespiratory fitness are associated with a higher increase in peak oxygen consumption (VO_2_peak) following an aerobic training intervention in MS patients.^5,6^ Reduction in fatigue following aerobic training has been observed only in MS patients experiencing significant baseline fatigue.^7^ Moreover, a multimodal exercise intervention showed greater benefits for MS patients with low baseline aerobic fitness, slow walking speed, and slow cognitive processing speed.^8^ Advanced MRI techniques may provide relevant information to better understand the mechanisms underlying the effects of specific rehabilitative approaches, contributing to explain the heterogeneity of responses. However, although some studies explored MRI predictors of response to cognitive rehabilitation,^9,10^ no study has investigated whether specific baseline MRI features may help predicting response to motor training.

The possibility to predict treatment response within two distinct rehabilitation approaches, such as aerobic and non-aerobic training, would help deliver prompt and tailored interventions.

This multiparametric study aimed to explore whether baseline demographic, clinical, structural, and functional MRI characteristics of MS patients, including lesional, volumetric, cortical thickness, diffusivity, and resting-state (RS) functional connectivity (FC) measures, may predict response to aerobic or non-aerobic training interventions.

## Materials and methods

This retrospective study was approved by the local ethical standards committee. Written informed consent was obtained from all participants.

### Study population

**Figure 1.**
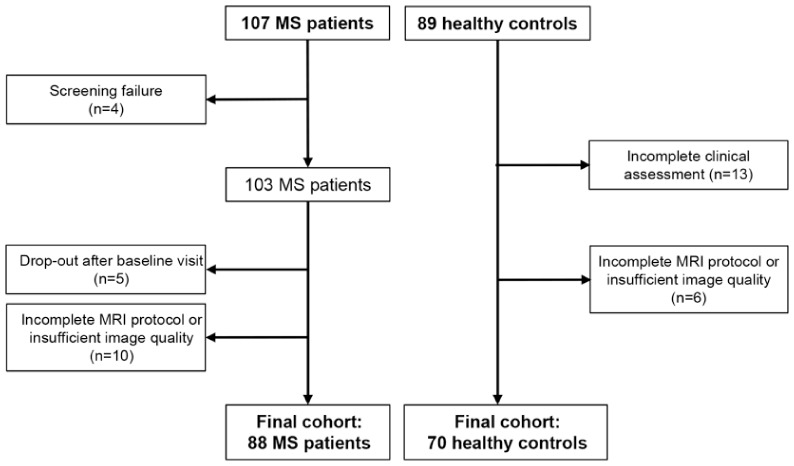
Flow diagram of the study selection process. Abbreviations: MRI = magnetic resonance imaging; MS = multiple sclerosis.

In this secondary analysis of an ongoing interventional rehabilitative protocol (ClinicalTrials.gov ID NCT04097418), we retrospectively analyzed 88 MS enrolled between 2016 and 2024. Seventy healthy controls (HC), recruited during the same period, were also analyzed to compare baseline measurements and determine potential pathological values (Figure 1). All MS patients and HC were required to meet these inclusion criteria: age between 18 and 65 years; right-handed^11^; no additional psychiatric, orthopedic, rheumatological, or neurological disorders beyond MS; no history of alcoholism or substance abuse; no regular aerobic exercise (> 1 day/week, > 30 minutes/session) in the 3 months before enrollment; no contraindications to cardiopulmonary exercise testing (CPET)^12^ or to MRI.

In addition, MS patients, diagnosed according to the 2017 revised McDonald criteria,^13^ had to be relapse- and steroid-free in the 3 months before enrollment, have a stable disease-modifying treatment (DMT) for MS from at least 6 months before enrollment, and an Expanded Disability Status Scale (EDSS) score ⩽ 6.5.

### Study design and interventions

MS patients were randomized, through a computer-generated sequence, to one of two groups: one undertaking an aerobic training program or another following a non-aerobic training regimen. Both groups completed 24 supervised 30–40-minute training sessions, distributed over 2–3 months, with continuous heart rate (HR) monitoring.

The aerobic training group performed moderate-intensity aerobic training on a recumbent arm–leg stepper, maintaining HR between 55% and 75% of their maximum HR as determined by CPET. The non-aerobic training group participated in a non-specific motor training program, including lower limb stretching, passive-active mobilization, and balance exercises.

### Clinical assessment and CPET

At baseline, MS patients underwent the 6-minute walking test (6MWT), which was repeated post-intervention to define response to training. MS patients were categorized as “responders” or “non-responders” based on a predefined threshold for responsiveness, defined as an improvement of at least 21.6 meters in the 6MWT. This threshold represents the anchor-based minimally important change (MIC) for MS patients.^14^

Baseline clinical assessment for both MS patients and HC also included Timed 25-Foot Walk Test (T25FWT) and Modified Fatigue Impact Scale (MFIS).

In addition, MS patients underwent a baseline complete neurologic evaluation, with definition of clinical phenotype, rating of the EDSS score,^15^ and recording of ongoing DMT.

For MS patients, baseline measurement of VO_2_peak was performed through CPET, employing a graded exercise protocol with progressively increasing intensity until exhaustion or the occurrence of limiting symptoms and/or signs.^16^ The VO_2_peak measure was considered reliable if two of the following four criteria were satisfied: (I) plateau in oxygen consumption despite increasing work rate, (II) peak respiratory exchange ratio ⩾ 1.10, (III) peak rated perceived exertion ⩾ 17, and (IV) peak HR within 10 beats of age-predicted maximum.^17^

### MRI acquisition

Using a 3.0 Tesla Philips Ingenia CX scanner (Philips Medical Systems, Eindhoven, The Netherlands) and standardized procedures for subjects positioning, the following brain MRI sequences were acquired from all subjects: (a) sagittal three-dimensional (3D) T1-weighted; (b) sagittal 3D fluid attenuation inversion recovery (FLAIR); (c) pulsed-gradient spin echo diffusion-weighted echo planar imaging (EPI); (d) T2*-weighted single-shot echo EPI sequence for RS fMRI. Details of the MRI protocol are available in Supplemental Material.

### MRI analysis

**Figure 2.**
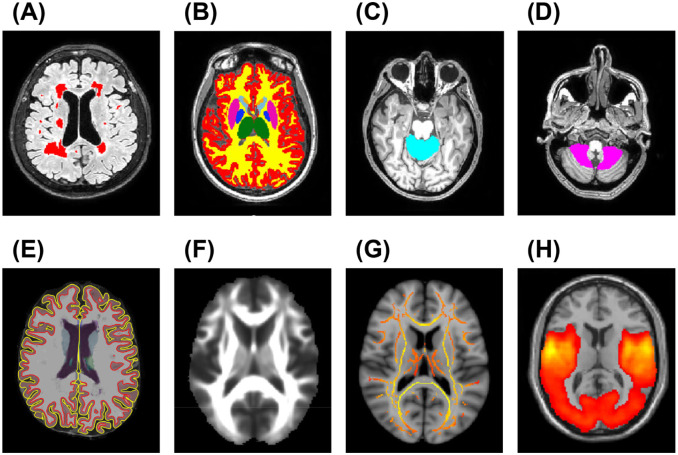
Examples of brain MRI analysis performed in this study. (a) Brain lesion segmentation (red-coded) on co-registered 3D FLAIR and 3D T1-weighted images; (b) segmentation of WM (yellow), cortex (red), and thalamus (green) on lesion-filled 3D T1-weighted images using SIENAx2; (c, d) segmentation of ACMA (light-blue) and PCMA (pink) on lesion-filled 3D T1-weighted images using SUIT; (e) WM surface (red) and pial surface (yellow) obtained using FreeSurfer 7.1.1. on lesion-filled 3D T1-weighted images for calculation of Cth; (f, g) mean FA image and WM tract skeleton obtained during DTI processing; (h) seed-based RS FC network of the left M1 in our cohort. Abbreviations: ACMA = anterior cerebellar motor area; Cth = cortical thickness; DTI = diffusion tensor imaging; FLAIR = fluid-attenuated inversion recovery; M1 = primary motor cortex; PCMA = posterior cerebellar motor area; RS FC = resting-state functional connectivity; WM = white matter.

Focal T2-hyperintense white matter (WM) lesions were identified by a fully automated approach using co-registered FLAIR and T1-weighted images as inputs. T2-hyperintense WM lesion volume (T2-LV) was obtained for each subject from the lesion mask, after visual inspection of the automated segmentation (Figure 2(a)).^18^

Normalized brain (NBV), cortical (NCV), WM (NWMV), and thalamic volumes were measured using FSL-SIENAx2 software on T1-weighted images after lesion filling (Figure 2(b)).^19^ The Spatially Unbiased Infratentorial Template (SUIT) was used to extract the volume of the anterior cerebellar motor area (ACMA), formed by bilateral lobules I-IV and lobule V, and the posterior cerebellar motor area (PCMA), formed by bilateral lobules VIIIA and VIIIB and their vermal portions (Figure 2(c) and (d)).^20^ All segmentations were visually inspected, and all volumes were corrected for head size, applying a scaling factor derived from FSL-SIENAx2.

The cross-sectional pipeline in FreeSurfer7.1.1^21^ was used on lesion-filled 3D T1-weighted images to assess cortical thickness (Cth) of the following areas, defined by the Human Brainnetome Atlas (http://atlas.brainnetome.org)^22^: primary motor cortex (M1), due to its crucial role in voluntary motor control; precuneus, anterior cingulate gyrus (ACG) and posterior cingulate gyrus (PCG), as key regions of the default mode network (DMN), which has been shown to be associated to severity of disability^23^ and to benefit from aerobic training (Figure 2(e)).^24^ We selected the superior temporal gyrus (STG) and middle temporal gyrus (MTG) as control regions to determine whether the informative role of volumetric measures in areas directly involved in motor functions was specific.

Tract-based spatial statistics analysis was performed to obtain WM fractional anisotropy (FA) and mean diffusivity (MD) from a priori selected WM tracts, as defined by the JHU combined atlas (Figure 2(f) and (g)),^25^ including the corticospinal tract (CST), middle cerebellar peduncle (MCP), and superior cerebellar peduncle (SCP), which are essential for motor functions. The superior longitudinal fasciculus (SLF) was included as control region.

Finally, a seed-based approach was used to assess mean global RS FC within the sensorimotor network (seeds: left and right M1), crucial for motor functions, and DMN (seed: bilateral precuneus), due to its link with disability severity^23^ and its association with aerobic training.^24^ Mean global RS FC within the auditory network (seed: left primary auditory cortex (A1) was evaluated as control network (Figure 2(h)).^26^

Details on MRI analysis are available in Supplemental Material.

### Statistical analysis

At baseline, differences in demographic and clinical variables between MS patients and HC, and between aerobic and non-aerobic groups, were tested using Chi-square, Welch’s t or Mann–Whitney tests, as appropriate. By using linear models in the HC group, we regressed out sex and age effects from inverse T25FWT scores. Residuals (i.e. difference between observed and predicted values) for all participants were scaled by HC error term thus obtaining z-scores.

Z-scores were similarly derived for baseline MRI variables, by fitting linear regression models to HC data. MRI sequence was included as an additional covariate for DTI and RS FC measures.

Brain T2-LV were log-transformed and compared between groups by a sex- and age-adjusted linear model. We run robust linear regression models^27^ to evaluate MRI z-scores in MS patients, as a whole and stratified according to the rehabilitation strategy, testing the null hypothesis that mean z-scores equal zero (i.e. healthy population expected value), and to compare them between the two patients’ groups. False discovery rate correction (Benjamini–Hochberg procedure) was applied to account for the overall number of tests.

In MS patients, the association of baseline variables with response to rehabilitative training was investigated by logistic regression models, using a robust quasi-likelihood-based estimation approach.^28^ Results were expressed as odds ratio in both training groups. Differences of association between training groups were assessed testing the significance of specific interaction terms. A training-adjusted odds ratio in the whole MS patients’ group was also computed. Since univariate analysis was intended for explorative purposes, no correction for multiplicity was performed.

Baseline predictors of training response were identified, in each training group and in the whole MS cohort, using the Boruta algorithm,^29^ which ranks features based on Random Forest variable importance while accounting for nonlinear effects and interactions and dealing with multicollinearity. To distinguish truly informative variables from those that could appear important by chance, Boruta compares each variable to “shadow” features—randomly permuted copies of the original variables. Selection stability and predictive performance were assessed using a 5-fold, 50-times repeated cross-validation (CV): Boruta was applied within each training fold, Random Forest models were fitted on the selected variables, and cross-validated area under the curve (CV-AUC) values were computed. The frequency of selection for each predictor across folds and repetitions was also recorded. Details of the Boruta feature selection and cross-validation procedures, along with the full list of baseline variables considered, are available in Supplemental Material.

SAS release 9.4 (SAS Institute, Cary, North Carolina) and Software *R* (version 4.3.3) were used for computations. *p*-values < 0.05 were deemed statistically significant.

## Results

### Demographic and clinical findings

Table 1 summarizes baseline demographic and clinical characteristics. Of the MS patients, 43 underwent aerobic training and 45 underwent non-aerobic training. Baseline demographic and clinical variables did not differ significantly between the two groups (*p* ⩾ 0.265).

**Table 1.** Baseline main demographic and clinical characteristics of healthy controls (HC) and multiple sclerosis (MS) patients, as a whole and stratified according to rehabilitative strategy (aerobic and non-aerobic group).

Variable	HC (70)	MS (88)	Aerobic (43)	Non-aerobic (45)	HC vs MS (*p-value*)	Aerobic vs Non-aerobic (*p-value*)
Sex: male/female (%)	23/47 (33%/67%)	31/57 (35%/65%)	16/27 (37%/63%)	15/30 (33%/67%)	0.886	0.875
Median age (IQR) [years]	48.32 (37.99;54.51)	49.86 (45.87;54.47)	49.42 (44.32;53.80)	51.33 (46.98;56.76)	0.160	0.265
Median EDSS (IQR)	-	4.0 (2.5;6.0)	3.5 (2.5;6.0)	4.5 (2.5;6.0)	-	0.590
Median disease duration (IQR) [years]	-	16.62 (9.14;25.12)	15.55 (9.62;26.68)	18.50 (9.00;24.00)	-	0.751
Phenotype: P/RR (%)	-	48/40 (55%/45%)	21/22 (49%/51%)	26/19 (58%/42%)	-	0.683
DMT: none/ME/HE^a^ (%)	-	22/28/38 (25%/32%/43%)	8/15/20 (19%/35%/46%)	14/13/18 (31%/29%/40%)	-	0.399
Mean 6MWT (SD) [meter]	-	347 (162)	346 (177)	347 (147)	-	0.980
Median T25FWT (IQR) [meter]	4.61 (4.15;5.25)	6.92 (5.33;10.62)	6.46 (5.26;10.88)	7.17 (5.34;10.45)	**<** **0.001**	0.733
Mean zT25FWT (SD)	0 (0.1)	-2.00 (1.87)	-2.07 (1.90)	-1.94 (1.86)		
Median VO_2_peak (IQR) [ml/Kg/min]	-	15.65 (12.20;18.93)	15.80 (12.60;18.95)	15.00 (12.20;17.90)	-	0.646
Median MFIS (IQR)	18 (12;27)	44 (27;51)	44 (27;53)	43 (27;51)	**<** **0.001**	0.534

Comparisons performed by Chi-square test (sex, phenotype, DMT), Welch’s *t*-test (6MWT, zT25FWT), and Mann–Whitney test (age, EDSS, disease duration, VO_2_peak, and MFIS).

a Classification of DMTs: ME = dimethyl fumarate, glatiramer acetate, interferon beta1a, teriflunomide; HE = alemtuzumab, azathioprine, cladribine, fingolimod, natalizumab, ocrelizumab, rituximab, siponimod.

Bold values denote statistical significance (*p* < 0.05).

Abbreviations. 6MWT = 6 minute walking test; DMT = disease-modifying treatment; EDSS = expanded disability status scale; HE = high efficacy; IQR = interquartile range; ME = moderate efficacy; MFIS = modified fatigue impact scale; P = progressive; RR = relapsing remitting; T25FWT = timed 25-foot walk test; VO_2_peak = peak oxygen consumption; zT25FWT = timed 25-foot walk test z-score.

Post intervention, 20 (47%) MS patients in the aerobic group were classified as responders, while 23 (53%) were non-responders. In the non-aerobic group, 14 (31%) MS patients were responders and 31 (69%) were non-responders. The difference in response rates between the two groups was not statistically significant (*p* = 0.206).

### Brain MRI findings

Table 2 summarizes brain structural and functional MRI features. Compared to HC, all MS patients had significantly lower NBV, NWMV, NCV, NTV, ACMA volume, PCMA volume, M1 Cth, precuneus Cth, PCG Cth, STG Cth, MTG Cth, MCP FA, SCP FA, CST FA, SLF FA, and mean global RS FC of left and right M1 (*p* ⩽ 0.004). In addition, MS patients had significantly higher brain T2-LV, MCP MD, SCP MD, CST MD, and SLF MD (*p* < 0.001). No significant differences were found in ACG Cth and mean global RS FC of the precuneus and left A1 (*p* ⩾ 0.052).

**Table 2.** Baseline MRI characteristics of multiple sclerosis (MS) patients, as a whole and stratified according to rehabilitative strategy (aerobic and non-aerobic group).

Variable	MS (88)		Aerobic (43)		Non-aerobic (45)		Aerobic vs Non-aerobic
	EM (SE)	*p* (FDR-p)	EM (SE)	*p* (FDR-p)	EM (SE)	*p* (FDR-p)	*p* (FDR-p)
T2-LV (ml)^a^	5.07 (0.67)	**<** **0.001** (< 0.001)	5.04 (0.94)	**<** **0.001** (< 0.001)	5.10 (0.92)	**<** **0.001** (< 0.001)	0.963 (0.963)
NBV	-2.35 (0.22)	**<** **0.001** (< 0.001)	-2.00 (0.31)	**<** **0.001** (< 0.001)	-2.66 (0.3)	**<** **0.001** (< 0.001)	0.131 (0.195)
NWMV	-2.03 (0.19)	**<** **0.001** (< 0.001)	-1.76 (0.28)	**<** **0.001** (< 0.001)	-2.25 (0.27)	**<** **0.001** (< 0.001)	0.208 (0.287)
NCV	-0.77 (0.13)	**<** **0.001** (< 0.001)	-0.64 (0.19)	**0.001** (0.002)	-0.88 (0.18)	**<** **0.001** (< 0.001)	0.359 (0.448)
NTV	-2.43 (0.22)	**<** **0.001** (< 0.001)	-2.29 (0.31)	**<** **0.001** (< 0.001)	-2.58 (0.30)	**<** **0.001** (< 0.001)	0.500 (0.589)
ACMA volume	-0.51 (0.13)	**<** **0.001** (< 0.001)	-0.49 (0.19)	**0.014** (0.025)	-0.53 (0.19)	**0.005** (0.010)	0.861 (0.905)
PCMA volume	-0.62 (0.15)	**<** **0.001** (< 0.001)	-0.65 (0.22)	**0.004** (0.009)	-0.60 (0.21)	**0.006** (0.012)	0.862 (0.905)
M1 Cth	-1.28 (0.19)	**<** **0.001** (< 0.001)	-1.27 (0.27)	**<** **0.001** (< 0.001)	-1.29 (0.27)	**<** **0.001** (< 0.001)	0.959 (0.963)
Precuneus Cth	-0.55 (0.15)	**<** **0.001** (0.001)	-0.54 (0.22)	**0.016** (0.029)	-0.56 (0.21)	**0.011** (0.021)	0.952 (0.963)
PCG Cth	-0.46 (0.14)	**0.002** (0.004)	-0.43 (0.21)	0.040 (0.070)	-0.50 (0.20)	**0.017** (0.029)	0.826 (0.885)
ACG Cth	-0.28 (0.13)	0.029 (0.052)	-0.33 (0.18)	0.073 (0.119)	-0.23 (0.18)	0.196 (0.278)	0.702 (0.776)
STG Cth	-0.81 (0.15)	**<** **0.001** (< 0.001)	-0.59 (0.21)	**0.006** (0.012)	-1.05 (0.21)	**<** **0.001** (< 0.001)	0.126 (0.191)
MTG Cth	-0.58 (0.15)	**<** **0.001** (< 0.001)	-0.43 (0.21)	0.041 (0.070)	-0.74 (0.21)	**<** **0.001** (0.001)	0.302 (0.396)
MCP FA	-2.31 (0.25)	**<** **0.001** (< 0.001)	-2.48 (0.36)	**<** **0.001** (< 0.001)	-2.15 (0.35)	**<** **0.001** (< 0.001)	0.513 (0.591)
SCP FA	-1.18 (0.16)	**<** **0.001** (< 0.001)	-1.31 (0.22)	**<** **0.001** (< 0.001)	-1.07 (0.21)	**<** **0.001** (< 0.001)	0.424 (0.506)
CST FA	-1.23 (0.18)	**<** **0.001** (< 0.001)	-1.47 (0.25)	**<** **0.001** (< 0.001)	-0.99 (0.25)	**<** **0.001** (< 0.001)	0.177 (0.255)
SLF FA	-0.88 (0.19)	**<** **0.001** (< 0.001)	-0.80 (0.27)	**0.003** (0.007)	-0.96 (0.26)	**<** **0.001** (< 0.001)	0.672 (0.758)
MCP MD	1.42 (0.21)	**<** **0.001(<** **0.001)**	1.65 (0.31)	**<** **0.001** (< 0.001)	1.24 (0.29)	**<** **0.001** (< 0.001)	0.330 (0.417)
SCP MD	0.89 (0.12)	**<** **0.001** (< 0.001)	1.05 (0.18)	**<** **0.001** (< 0.001)	0.75 (0.17)	**<** **0.001** (< 0.001)	0.230 (0.313)
CST MD	2.23 (0.24)	**<** **0.001** (< 0.001)	2.32 (0.34)	**<** **0.001** (< 0.001)	2.16 (0.34)	**<** **0.001** (< 0.001)	0.741 (0.802)
SLF MD	2.19 (0.24)	**<** **0.001** (< 0.001)	1.99 (0.35)	**<** **0.001** (< 0.001)	2.39 (0.34)	**<** **0.001** (< 0.001)	0.413 (0.499)
Left M1 RS FC^b^	-0.38 (0.09)	**<** **0.001** (< 0.001)	-0.25 (0.13)	0.064 (0.107)	-0.51 (0.13)	**<** **0.001** (< 0.001)	0.151 (0.221)
Right M1 RS FC^b^	-0.34 (0.10)	**<** **0.001** (0.001)	-0.17 (0.13)	0.201 (0.281)	-0.50 (0.13)	**<** **0.001** (< 0.001)	0.080 (0.130)
Precuneus RS FC^b^	0.07 (0.10)	0.529 (0.604)	0.23 (0.15)	0.124 (0.191)	-0.10 (0.15)	0.505 (0.589)	0.118 (0.188)
Left A1 RS FC^b^	-0.10 (0.10)	0.306 (0.396)	0.01 (0.14)	0.944 (0.963)	-0.21 (0.14)	0.132 (0.195)	0.268 (0.356)

Mean estimated z-scores, related standard errors, and p-values testing the null hypothesis that mean z-score equals zero (i.e. healthy population expected value) in MS patients, as a whole and according to training group. P-values of between-group comparisons are also provided. Analyses were performed using robust linear regression models. T2-hyperintense lesion volumes were compared by a sex- and age-adjusted linear model. FDR correction (Benjamini–Hochberg procedure) was applied to account for the overall number of tests performed.

Bold values denote statistical significance (*p* < 0.05).

Abbreviations. A1 = primary auditory cortex; ACG = anterior cingulate gyrus; ACMA = anterior cerebellar motor area; CST = corticospinal tract; Cth = cortical thickness; EM = estimated mean; FA = fractional anisotropy; FDR = false discovery rate; M1 = primary motor cortex; MCP = middle cerebellar peduncle; MD = mean diffusivity; MTG = middle temporal gyrus; NBV = normalized brain volume; NCV = normalize cortical volume; NTV = normalize thalamic volume; NWMV = normalize white matter volume; PCG = posterior cingulate gyrus; PCMA = posterior cerebellar motor area; RS FC = resting-state functional connectivity; SCP = superior cerebellar peduncle; SE = standard error; SLF = superior longitudinal fasciculus; STG = superior temporal gyrus; T2-LV = T2-hyperintense lesion volume.

a Analysis performed on log-transformed data. Estimated means and standard errors reported are back-transformed on the original scale using delta method.

b Mean global RS FC with the remaining brain regions.

Similar results were observed when the aerobic and non-aerobic training MS groups were separately compared to HC.

There were no differences in any of the assessed MRI variables between the aerobic and non-aerobic training groups (*p* ⩾ 0.130).

### Univariate analysis

The results of univariate analyses are shown in Table 3. Response to training was associated with higher MCP FA (odds ratio (OR) = 1.03, 95% confidence interval (CI) = 1.00;1.07, *p* = 0.049) and CST FA (OR = 1.06, 95% CI = 1.01;1.11, *p* = 0.027) in the aerobic group and with lower SCP MD (OR = 0.89, 95% CI = 0.81;0.98, *p* = 0.017) in the non-aerobic group.

**Table 3.** Univariate associations of demographic, clinical and MRI variables with response to training in the aerobic group, non-aerobic group and in the whole cohort of MS patients.

		Aerobic		Non-aerobic		Aerobic *vs* Non-aerobic	Whole MS cohort	
		OR (95% CI)	*p*	OR (95% CI)	*p*	*P*	OR (95% CI)	*p*
Sex (male *vs* female)		0.56 (0.16;1.97)	0.364	0.73 (0.18;2.88)	0.650	0.780	0.62 (0.24;1.59)	0.322
Age^a^		1.30 (0.57;2.92)	0.533	0.93 (0.40;2.19)	0.875	0.586	1.12 (0.62;2.03)	0.697
EDSS^b^		0.88 (0.64;1.22)	0.450	0.97 (0.69;1.37)	0.870	0.688	0.92 (0.73;1.16)	0.486
Disease duration^b^		0.99 (0.94;1.05)	0.749	0.97 (0.90;1.03)	0.337	0.599	0.98 (0.94;1.03)	0.406
Phenotype (P *vs* RR)		0.43 (0.13;1.46)	0.175	0.96 (0.27;3.46)	0.954	0.370	0.62 (0.26;1.49)	0.288
DMT	ME vs. none	0.53 (0.09;3.03)	0.568	2.14 (0.44;10.55)	0.368	0.488	1.11 (0.35;3.53)	0.436
	HE vs. none	0.40 (0.07;2.16)		0.71 (0.14;3.60)			0.59 (0.19;1.82)	
	HE *vs* ME	0.76 (0.20;2.95)		0.33 (0.07;1.59)			0.53 (0.19;1.48)	
6MWT^a^		1.01 (0.98;1.05)	0.448	1.02 (0.97;1.06)	0.451	0.892	1.01 (0.99;1.04)	0.295
zT25FWT^c^		1.02 (0.98;1.05)	0.292	1.00 (0.97;1.04)	0.835	0.560	1.01 (0.99;1.04)	0.326
MFIS^b^		1.02 (0.98;1.05)	0.381	0.98 (0.94;1.01)	0.228	0.140	1.00 (0.97;1.02)	0.790
VO_2_peak^d^		0.80 (0.40;1.57)	0.512	1.04 (0.56;1.95)	0.898	0.569	0.92 (0.58;1.45)	0.712
T2-LV^e^		1.01 (0.98;1.04)	0.259	0.96 (0.91;1.01)	0.134	0.713	0.97 (0.93;1.00)	0.067
NBV^c^		1.01 (0.98;1.04)	0.478	1.03 (0.99;1.06)	0.182	0.560	1.02 (0.99;1.04)	0.164
NWMV^c^		1.00 (0.97;1.03)	0.883	1.01 (0.97;1.05)	0.635	0.639	1.00 (0.98;1.03)	0.911
NCV^c^		1.03 (0.98;1.08)	0.221	1.03 (0.97;1.10)	0.262	0.912	1.03 (0.99;1.07)	0.097
NTV^c^		1.02 (0.98;1.05)	0.340	1.02 (0.98;1.05)	0.310	0.949	1.02 (0.99;1.04)	0.166
ACMA volume^c^		0.99 (0.96;1.03)	0.737	1.03 (0.98;1.10)	0.247	0.249	1.01 (0.97;1.04)	0.732
PCMA volume^c^		1.01 (0.97;1.05)	0.669	1.02 (0.97;1.08)	0.406	0.662	1.01 (0.98;1.05)	0.396
M1 Cth^c^		1.01 (0.97;1.04)	0.747	1.00 (0.97;1.04)	0.803	0.954	1.00 (0.98;1.03)	0.688
Precuneus Cth^c^		1.00 (0.96;1.04)	0.984	0.99 (0.95;1.04)	0.800	0.840	1.00 (0.97;1.03)	0.875
PCG Cth^c^		1.02 (0.97;1.07)	0.526	1.03 (0.98;1.08)	0.304	0.759	1.02 (0.99;1.06)	0.245
ACG Cth^c^		0.98 (0.93;1.04)	0.569	1.01 (0.96;1.07)	0.713	0.508	1.00 (0.96;1.04)	0.897
STG Cth^c^		1.00 (0.96;1.04)	0.989	1.00 (0.97;1.04)	0.844	0.884	1.00 (0.97;1.03)	0.886
MTG Cth^c^		0.98 (0.93;1.03)	0.374	1.00 (0.96;1.04)	0.905	0.435	0.99 (0.96;1.02)	0.649
MCP FA^c^		1.03 (1.00;1.07)	**0.049**	1.02 (0.99;1.06)	0.131	0.717	1.03 (1.01;1.05)	**0.012**
SCP FA^c^		1.03 (0.99;1.07)	0.159	1.05 (0.99;1.12)	0.098	0.531	1.03 (1.00;1.07)	**0.041**
CST FA^c^		1.06 (1.01;1.11)	**0.027**	1.01 (0.97;1.05)	0.628	0.156	1.03 (1.00;1.06)	0.057
SLF FA^c^		1.04 (1.00;1.08)	0.073	1.01 (0.97;1.05)	0.592	0.307	1.03 (1.00;1.06)	0.061
MCP MD^c^		0.97 (0.94;1.00)	0.075	0.96 (0.92;1.01)	0.098	0.726	0.97 (0.94;0.99)	**0.016**
SCP MD^c^		0.95 (0.89;1.01)	0.075	0.89 (0.81;0.98)	**0.017**	0.274	0.93 (0.88;0.97)	**0.003**
CST MD^c^		0.98 (0.95;1.01)	0.127	0.98 (0.94;1.01)	0.192	0.976	0.98 (0.96;1.00)	**0.045**
SLF MD^c^		0.99 (0.96;1.01)	0.246	0.99 (0.96;1.02)	0.453	0.825	0.99 (0.97;1.01)	0.162
Left M1 RS FC^c^		1.05 (0.97;1.13)	0.246	1.04 (0.97;1.12)	0.238	0.999	1.04 (0.99;1.1)	0.098
Right M1 RS FC^c^		1.04 (0.97;1.12)	0.274	1.04 (0.97;1.11)	0.305	0.944	1.04 (0.99;1.09)	0.133
Precuneus RS FC^c^		1.02 (0.95;1.10)	0.509	1.05 (0.98;1.14)	0.162	0.572	1.04 (0.99;1.09)	0.147
Left A1 RS FC^c^		1.02 (0.95;1.09)	0.570	1.02 (0.95;1.10)	0.533	0.944	1.02 (0.97;1.07)	0.404

Bold values denote statistical significance (*p* < 0.05).

Abbreviations. 6MWT = 6 minute walking test; ACG = anterior cingulate gyrus; ACMA = anterior cerebellar motor area; CI = confidence interval; CST = corticospinal tract; Cth = cortical thickness; DMT = disease-modifying treatments; EDSS = expanded disability status scale; EM = estimated mean; FA = fractional anisotropy; FDR = false discovery rate; HE = high efficacy; M1 = primary motor cortex; MCP = middle cerebellar peduncle; MD = mean diffusivity; ME = moderate efficacy; MFIS = modified fatigue impact scale; MTG = middle temporal gyrus; NBV = normalized brain volume; NCV = normalize cortical volume; NTV = normalize thalamic volume; NWMV = normalize white matter volume; OR = odds ratio; *p* = progressive; PCG = posterior cingulate gyrus; PCMA = posterior cerebellar motor area; RR = relapsing remitting; RS FC = resting-state functional connectivity; SCP = superior cerebellar peduncle; SE = standard error; SLF = superior longitudinal fasciculus; STG = superior temporal gyrus; T25FWT = timed 25-foot walk test; T2-LV = T2-hyperintense lesion volume; VO_2_peak = peak oxygen consumption.

a OR associated with a 10-unit increase in the predictor.

b OR associated with a 1-unit increase in the predictor.

c OR associated with a 0.1-z-score increase in the predictor.

d OR associated with a 5-unit increase in the predictor.

e OR associated with a 10%-increase in the predictor.

No differences were observed in the associations between the assessed variables and response to training when comparing the two training groups (*p* ⩾ 0.140).

In the whole cohort of MS patients, response to training was associated with higher MCP FA (OR = 1.03, 95% CI = 1.01;1.05, *p* = 0.012), higher SCP FA (OR = 1.03, 95% CI = 1.01;1.07, *p* = 0.041), lower MCP MD (OR = 0.97, 95% CI = 0.94;0.99, *p* = 0.016), lower SCP MD (OR = 0.93, 95% CI = 0.88;0.97, *p* = 0.003), and lower CST MD (OR = 0.98, 95% CI = 0.96;1.00, *p* = 0.045). None of the remaining variables showed significant associations with response to training (*p* ⩾ 0.057).

### Random forest and cross-validation analyses

**Figure 3.**
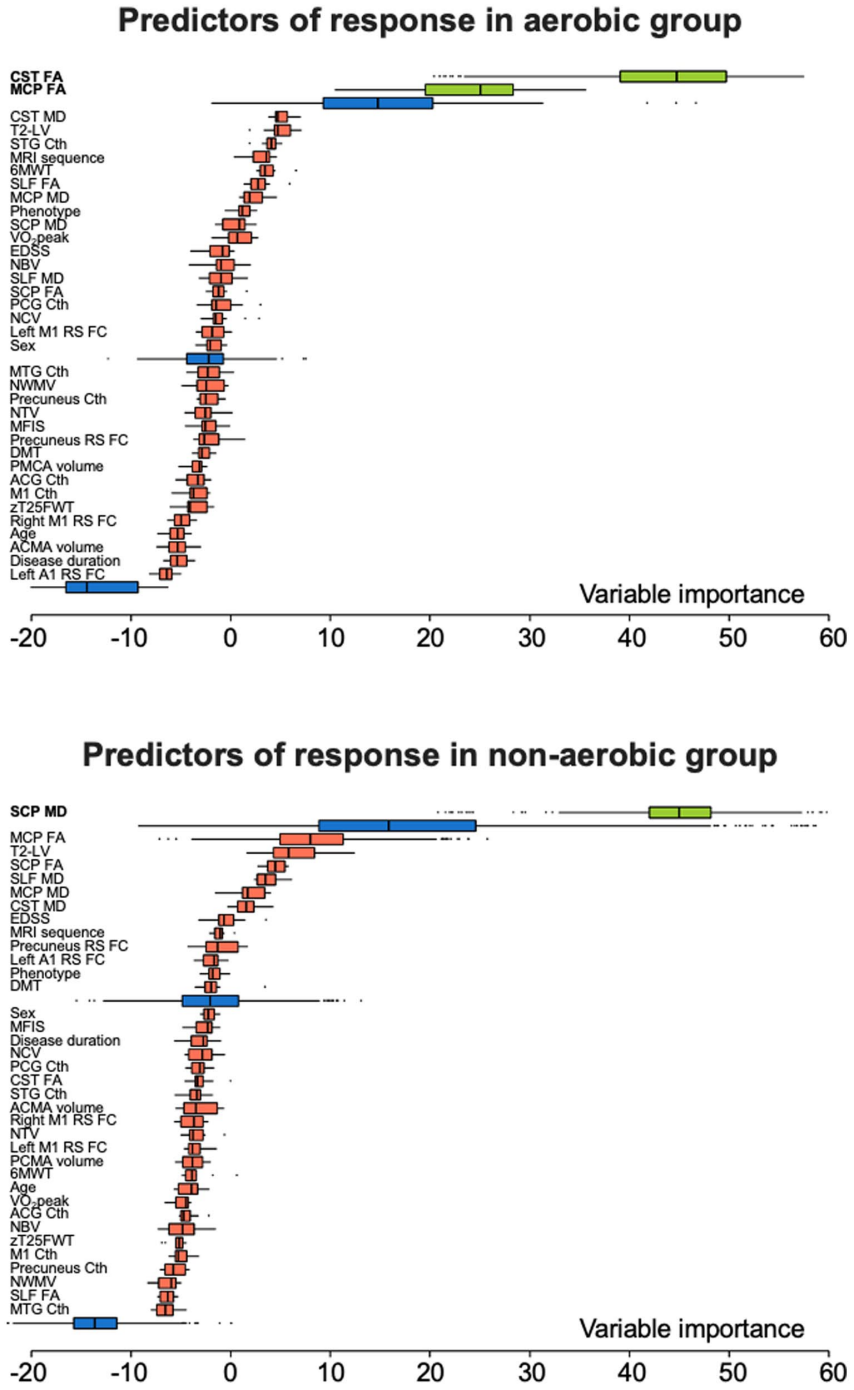
Random forest informative predictors of response to aerobic training and non-aerobic training. Distribution of variable importance, achieved across iterations of Boruta algorithm of demographic, clinical and MRI features to explain response to training in the aerobic and non-aerobic group. Boruta compares the importance of the original variables with the highest feature importance of the shadow features, obtained using feature permuted copies. Poorly performing variables are progressively discarded. Selected features are shown in green, discarded features in red. Maximum, mean, and minimum importance achieved by shadow attributes are shown in blue. Abbreviations. 6MWT = 6 minute walking test; A1 = primary auditory cortex; ACG = anterior cingulate gyrus; ACMA = anterior cerebellar motor area; CST = corticospinal tract; Cth = cortical thickness; DMT = disease-modifying treatment; EDSS = expanded disability status scale; FA = fractional anisotropy; M1 = primary motor cortex; MCP = middle cerebellar peduncle; MD = mean diffusivity; MFIS = modified fatigue impact scale; MTG = middle temporal gyrus; NBV = normalized brain volume; NCV = normalize cortical volume; NTV = normalize thalamic volume; NWMV = normalize white matter volume; PCG = posterior cingulate gyrus; PCMA = posterior cerebellar motor area; RS FC = resting-state functional connectivity; SCP = superior cerebellar peduncle; SLF = superior longitudinal fasciculus; STG = superior temporal gyrus; T2-LV = T2-hyperintense lesion volume; VO_2_peak = peak oxygen consumption; zT25FWT = timed 25-foot walk test z-score.

**Figure 4.**
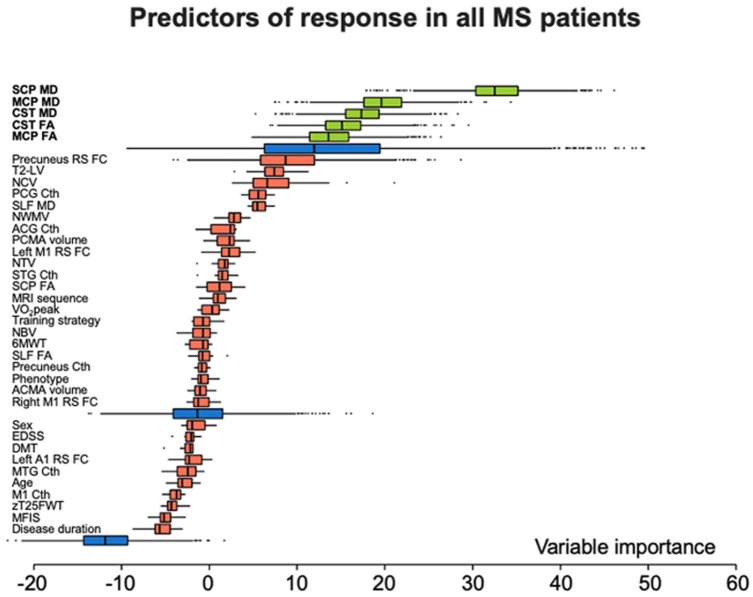
Random forest informative predictors of response to physical exercise. Distribution of variable importance, achieved across iterations of Boruta algorithm of demographic, clinical, and MRI features to explain response to training in all MS patients independently of the training strategy proposed. Boruta compares the importance of the original variables with the highest feature importance of the shadow features, obtained using feature permuted copies. Poorly performing variables are progressively discarded. Selected features are shown in green, discarded features in red. Maximum, mean, and minimum importance achieved by shadow attributes are shown in blue. Abbreviations. 6MWT = 6 minute walking test; A1 = primary auditory cortex; ACG = anterior cingulate gyrus; ACMA = anterior cerebellar motor area; CST = corticospinal tract; Cth = cortical thickness; DMT = disease-modifying treatment; EDSS = expanded disability status scale; FA = fractional anisotropy; M1 = primary motor cortex; MCP = middle cerebellar peduncle; MD = mean diffusivity; MFIS = modified fatigue impact scale; MTG = middle temporal gyrus; NBV = normalized brain volume; NCV = normalize cortical volume; NTV = normalize thalamic volume; NWMV = normalize white matter volume; PCG = posterior cingulate gyrus; PCMA = posterior cerebellar motor area; RS FC = resting-state functional connectivity; SCP = superior cerebellar peduncle; SLF = superior longitudinal fasciculus; STG = superior temporal gyrus; T2-LV = T2-hyperintense lesion volume; VO_2_peak = peak oxygen consumption; zT25FWT = timed 25-foot walk test z-score.

In the aerobic group, informative predictors of response to training were, in order of importance, CST FA (relative importance (RI) and MCP FA (RI = 56.0). In the non-aerobic training group, the only predictor of response was SCP MD (RI = 100). In the whole cohort of MS patients, informative predictors of response to training were, in order of importance, SCP MD (RI = 100), MCP MD (RI = 60.3), CST MD (RI = 53.3), CST FA (RI = 46.5), and MCP FA (RI = 41.8) (Table 4; Figures 3 and 4). The predictor selection frequencies observed in the repeated cross-validation analysis mirrored the global variable importance obtained from the full sample in the aerobic group (median CV-AUC = 0.648, IQR = 0.583;0.687), in the non-aerobic training group (median CV-AUC = 0.672; IQR = 0.645;0.716) and in the whole MS cohort (median CV-AUC = 0.665, IQR = 0.644;0.685) (Table 4 and Supplementary Figure 1).

**Table 4.** Random forest informative predictors of response to training, identified using the Boruta algorithm, in the aerobic group, the non-aerobic group and in all MS patients.

Group	Variable	Median Importance (IQR)	Relative Importance	CV selection percentage (%)	Median CV-AUC (IQR)
Aerobic group (*n* = 20/43)	CST FA	44.80 (39.1;49.7)	100	94.4	0.648 (0.583;0.687)
	MCP FA	25.1 (19.5;28.3)	56.0	55.6	
Non-aerobic group (*n* = 14/45)	SCP MD	45.0 (42.0;48.2)	100	94.0	0.672 (0.645;0.716)
All MS patients (*n* = 34/88)	SCP MD	32.6 (30.3;35.1)	100	90.8	0.665 (0.644;0.685)
	MCP MD	19.6 (17.6;21.9)	60.3	56.0	
	CST MD	17.3 (15.5;19.3)	53.3	48.8	
	CST FA	15.1 (13.2;17.2)	46.5	39.6	
	MCP FA	13.6 (11.4;15.9)	41.8	39.2	
	Precuneus RS FC	-	-	34.8	

The table reports response predictors to training, selected using the Boruta algorithm and ranked by median importance across Boruta iterations, in the aerobic group, the non-aerobic group, and in all MS patients. Results from cross-validation are also summarized: in 50-times repeated 5-fold cross-validation (CV), Boruta was applied within each training fold to select predictors, and a random forest model was fitted on them. Predictions from the held-out folds were pooled within each repetition to calculate CV-AUC. Selection percentages indicate how often a predictor was retained across all training folds and repetitions (only predictors selected in at least 30% of runs are shown).

Abbreviations. AUC = area under the curve; CST = corticospinal tract; CV = cross-validation; FA = fractional anisotropy; IQR = interquartile range; MCP = middle cerebellar peduncle; MD = mean diffusivity; MS = multiple sclerosis; *n* = number of responders/total group patients; RS FC = resting-state functional connectivity; SCP = superior cerebellar peduncle.

## Discussion

This study aimed to examine whether baseline characteristics of MS patients may predict response to physical rehabilitation. Specifically, we sought to identify potential predictors of improvement in the 6MWT among demographic, clinical, structural, and functional MRI features following aerobic or non-aerobic training regimens. The 6MWT was chosen since it measures walking endurance, a critical limiting factor in the everyday life of MS patients,^30^ and it encompasses different motor components that may be improved by both training strategies. Regardless of the training strategy, response to training was associated with specific measures of microstructural damage in motor-related WM tracts.

Consistent with the literature,^31^ MS patients showed focal WM lesions, WM microstructural abnormalities and atrophy compared to HC. Conversely, except for M1, functional networks did not differ, reflecting the occurrence of functional network abnormalities in response to brain damage that may be heterogeneous among MS patients in the different disease phases. While structural damage accumulates progressively, RS FC is more dynamic. Early in the disease, it may be preserved or temporarily increased to compensate for structural damage. As the disease advances, these mechanisms collapse, resulting in maladaptive RS FC abnormalities.^32^

Specific predictors of response to training were identified in both groups using univariate and random forest analyses.

In the aerobic group, response to rehabilitative training was predicted by higher FA in the CST and MCP, both crucial WM tracts for motor functions. Besides improving cardiorespiratory fitness, aerobic training may promote neuronal oxygenation and the production of brain-derived neurotrophic factor (BDNF).^33^ BDNF supports neuroplasticity by strengthening neuronal connections, aiding neural repair, and promoting neurogenesis.^33^ Although further studies are needed to confirm our findings, we could hypothesize that WM tracts with higher integrity may represent optimal targets for BDNF and other neuroplasticity-promoting factors to exert their beneficial effects, while more damaged WM tracts may limit the potential benefits of this rehabilitative strategy. This hypothesis aligns with previous findings indicating that better responses to cognitive rehabilitation were predicted by lower structural network disruption between regions associated with the DMN.^10^

In the non-aerobic group, the only predictor of training response was lower SCP MD, consistent with the balance-focused intervention. Given the cerebellum’s role in motor regulation, SCP’s integrity may enhance training benefits, whereas its damage could impair the cerebellar capacity to adjust and refine movements. Notably, training-induced balance improvement in MS patients was found to be associated with dynamic microstructural changes in the SCP, reinforcing our findings and suggesting behavior-dependent modulation of myelin and axons in this structure.^34^

Given the lack of significant differences between the aerobic and non-aerobic groups in the associations between baseline features and response to training, we performed additional analyses considering all MS patients as a combined group to strengthen the robustness of our findings and assess predictors of response to motor exercise more in general. This approach identified a broader set of predictors, all of which were measures of microstructural damage in WM tracts associated with motor function. Although we did not explore whether clinical improvements were associated with MRI changes, it is possible that less severe microstructural damage of WM tracts may enable greater brain plasticity and adaptive changes in response to training. This hypothesis is supported by previous studies highlighting mechanisms such as axon branching, sprouting, changes in myelination, and astrocyte activity as critical to functional recovery.^35^

It is worth noting that the predictors most frequently selected during cross-validation largely aligned with the global importance rankings derived from the full dataset. Despite the moderate overall discriminative performance, as reflected by the CV-AUC values, this reproducibility across resampling iterations supports the stability of feature selection, confirming the robustness of the procedure.

Taken together, our findings highlight the specificity of WM tract microstructural integrity in predicting rehabilitative outcomes, with a particular relevance for WM tracts directly involved in motor functions.

No RS FC-derived measure predicted response to motor training, unlike previous findings for cognitive rehabilitation.^9^ This difference may reflect the distinct nature of cognitive and motor functions. While cognitive processes rely on complex networks involving multiple brain regions, motor function primarily depends on the integrity of motor pathways.

This study has several limitations. First, the sample size was relatively small. A larger sample size in both training groups might have clarified distinctions between the predictors of response to the two training strategies. Moreover, since the effects of rehabilitation may differ at different disease stages, stratifying analyses by disability level in future studies could yield more targeted insights. We defined improvement in the 6MWT using a threshold based on an anchor-based MIC for MS patients identified in a well-powered multicenter study.^14^ While this is a robust estimate, alternative thresholds derived from different methods may generate different results. We did not investigate longitudinal MRI changes between responders and non-responders. Future studies could address this by assessing whether such analyses reveal differential brain changes associated with treatment response. Finally, we did not include a longer follow-up assessment following rehabilitation training.

In conclusion, response to motor training in MS patients is associated with microstructural integrity in key WM tracts related to motor function, independent of the rehabilitative strategy proposed. These findings highlight the potential for advanced neuroimaging measures to identify MS patients most likely to benefit from motor rehabilitation and provide insights into its underlying mechanisms. Furthermore, the association between training effectiveness and preserved WM integrity suggests that early rehabilitation may help maximize outcomes.

## Supplemental Material

sj-docx-1-msj-10.1177_13524585251398386 – Supplemental material for Contribution of structural and functional MRI in predicting response to motor training in multiple sclerosis Supplemental material, sj-docx-1-msj-10.1177_13524585251398386 for Contribution of structural and functional MRI in predicting response to motor training in multiple sclerosis by Tetsu Morozumi, Paolo Preziosa, Alessandro Meani, Elisabetta Pagani, Paola Valsasina, Chiara Arezzo, Francesco Romanò, Matteo Albergoni, Massimo Filippi and Maria A Rocca in Multiple Sclerosis Journal

sj-docx-2-msj-10.1177_13524585251398386 – Supplemental material for Contribution of structural and functional MRI in predicting response to motor training in multiple sclerosis Supplemental material, sj-docx-2-msj-10.1177_13524585251398386 for Contribution of structural and functional MRI in predicting response to motor training in multiple sclerosis by Tetsu Morozumi, Paolo Preziosa, Alessandro Meani, Elisabetta Pagani, Paola Valsasina, Chiara Arezzo, Francesco Romanò, Matteo Albergoni, Massimo Filippi and Maria A Rocca in Multiple Sclerosis Journal

sj-pdf-3-msj-10.1177_13524585251398386 – Supplemental material for Contribution of structural and functional MRI in predicting response to motor training in multiple sclerosis Supplemental material, sj-pdf-3-msj-10.1177_13524585251398386 for Contribution of structural and functional MRI in predicting response to motor training in multiple sclerosis by Tetsu Morozumi, Paolo Preziosa, Alessandro Meani, Elisabetta Pagani, Paola Valsasina, Chiara Arezzo, Francesco Romanò, Matteo Albergoni, Massimo Filippi and Maria A Rocca in Multiple Sclerosis Journal
