# Supplementary material for: Contribution of structural and functional MRI in predicting response to motor training in multiple sclerosis
Source: Mult Scler. 2025 Dec 19;32(1):93–106. doi: 10.1177/13524585251398386 (PMC12756515; doi:10.1177/13524585251398386)
Supplement: sj-docx-1-msj-10.1177_13524585251398386 – Supplemental material for Contribution of structural and functional MRI in predicting response to motor training in multiple sclerosis [file sj-docx-1-msj-10.1177_13524585251398386.docx]

MS Journal Appendix for MRI methodology

| **Hardware** | |
| --- | --- |
| Field strength | 3.0 T |
| Manufacturer | Philips Medical Systems |
| Model | Philips Ingenia CX |
| Coil type  (e.g. head, surface) | dS-Head-32 |
| Number of coil channels | 32 |

| **Acquisition sequence** | | |
| --- | --- | --- |
| Type  (e.g. FLAIR, DIR, DTI, fMRI) | FLAIR | |
| Acquisition time | 6.15 min | |
| Orientation | Sagittal | |
| Alignment  (e.g. anterior commissure/poster commissure line) | None | |
| Voxel size | 1x1x1 mm | |
| TR | 4800 ms | |
| TE | 270 ms | |
| TI | 1650 ms | |
| Flip angle | Variable flip angle | |
| NEX | 2 | |
| Field of view | 256x256 mm | |
| Matrix size | 256x256 | |
| Parallel imaging | Yes | No |
| If used, parallel imaging method:  (e.g. SENSE, GRAPPA) | SENSE | |
| Cardiac gating | Yes | No |
| If used, cardiac gating method:  (e.g. PPU or ECG) |  | |
| Contrast enhancement | Yes | No |

| **Acquisition sequence** | | |
| --- | --- | --- |
| Type  (e.g. FLAIR, DIR, DTI, fMRI) | MPRAGE | |
| Acquisition time | 8.53 min | |
| Orientation | Sagittal | |
| Alignment  (e.g. anterior commissure/poster commissure line) | None | |
| Voxel size | 1x1x1 mm | |
| TR | 7 ms | |
| TE | 3.2 ms | |
| TI | 1000 ms | |
| Flip angle | 8° | |
| NEX | 1 | |
| Field of view | 256x256 mm | |
| Matrix size | 256x256 | |
| Parallel imaging | Yes | No |
| If used, parallel imaging method:  (e.g. SENSE, GRAPPA) | N.A. | |
| Cardiac gating | Yes | No |
| If used, cardiac gating method:  (e.g. PPU or ECG) |  | |
| Contrast enhancement | Yes | No |

| **Acquisition sequence** | | |
| --- | --- | --- |
| Type  (e.g. FLAIR, DIR, DTI, fMRI) | DTI (1) | |
| Acquisition time | 11 min | |
| Orientation | Axial | |
| Alignment  (e.g. anterior commissure/poster commissure line) | None | |
| Voxel size | 2.14x2.71x2.3 mm | |
| TR | 8775 ms | |
| TE | 58 ms | |
| TI | - | |
| Flip angle | 90 | |
| NEX | 2 | |
| Field of view | 240x231 mm | |
| Matrix size | 112x88 | |
| Parallel imaging | Yes | No |
| If used, parallel imaging method:  (e.g. SENSE, GRAPPA) | SENSE | |
| Cardiac gating | Yes | No |
| If used, cardiac gating method:  (e.g. PPU or ECG) |  | |
| Contrast enhancement | Yes | No |

| **Acquisition sequence** | | |
| --- | --- | --- |
| Type  (e.g. FLAIR, DIR, DTI, fMRI) | fMRI (1) | |
| Acquisition time | 10 min | |
| Orientation | axial | |
| Alignment  (e.g. anterior commissure/poster commissure line) | None | |
| Voxel size | 2x2x4 mm | |
| TR | 3000 ms | |
| TE | 35 ms | |
| TI | - | |
| Flip angle | 85° | |
| NEX | 1 | |
| Field of view | 240x240 | |
| Matrix size | 128x128 | |
| Parallel imaging | Yes | No |
| If used, parallel imaging method:  (e.g. SENSE, GRAPPA) | SENSE | |
| Cardiac gating | Yes | No |
| If used, cardiac gating method:  (e.g. PPU or ECG) |  | |
| Contrast enhancement | Yes | No |

| **Acquisition sequence** | | |
| --- | --- | --- |
| Type  (e.g. FLAIR, DIR, DTI, fMRI) | DTI (2) | |
| Acquisition time | 11.5 min | |
| Orientation | Axial | |
| Alignment  (e.g. anterior commissure/poster commissure line) | None | |
| Voxel size | 2.14x2.69x2.3 mm | |
| TR | 5900 ms | |
| TE | 78 ms | |
| TI | - | |
| Flip angle | 90 | |
| NEX | 1 | |
| Field of view | 240x233 mm | |
| Matrix size | 112x85 | |
| Parallel imaging | Yes | No |
| If used, parallel imaging method:  (e.g. SENSE, GRAPPA) | SENSE | |
| Cardiac gating | Yes | No |
| If used, cardiac gating method:  (e.g. PPU or ECG) |  | |
| Contrast enhancement | Yes | No |

| **Acquisition sequence** | | |
| --- | --- | --- |
| Type  (e.g. FLAIR, DIR, DTI, fMRI) | fMRI (2) | |
| Acquisition time | 8 min | |
| Orientation | Axial | |
| Alignment  (e.g. anterior commissure/poster commissure line) | None | |
| Voxel size | 2.5x2.5x3 mm | |
| TR | 1560 ms | |
| TE | 35 ms | |
| TI | - | |
| Flip angle | 70° | |
| NEX | 1 | |
| Field of view | 240x240 mm | |
| Matrix size | 96x96 | |
| Parallel imaging | Yes | No |
| If used, parallel imaging method:  (e.g. SENSE, GRAPPA) | SENSE | |
| Cardiac gating | Yes | No |
| If used, cardiac gating method:  (e.g. PPU or ECG) |  | |
| Contrast enhancement | Yes | No |

| **Image analysis methods and outputs** | |
| --- | --- |
| ***Brain lesions*** | |
| Type  (e.g. Gd-enhancing, T2-hyperintense, T1-hypointense) | T2-hyperintense |
| Analysis method | Fully automated deep-learning method |
| Analysis software | nicMSlesions (Valverde et al., 2017) |
| Output measure  (e.g. count or volume [ml]) | Volume [ml] |
| ***Brain tissue volumes*** | |
| Type  (e.g. whole brain, grey matter, white matter, spinal cord) | Whole brain, cortical, white matter, thalamus, anterior cerebellar motor area and posterior cerebellar motor area |
| Analysis method | Fully automated tissue segmentation |
| Analysis software | FSL-SIENAx2, FIRST, SUIT |
| Output measure  (e.g. absolute tissue volume in ml, tissue volume as a fraction of intracranial volume, percentage change in tissue volumes) | Tissue volume in ml normalized for head size |
| ***Cortical thickness*** | |
| Type  (e.g. whole brain, grey matter, white matter, spinal cord) | Primary motor cortices, precuneus, anterior cingulate gyrus, posterior cingulate gyrus, superior temporal gyrus and middle temporal gyrus defined by Human Brainettome Atlas |
| Analysis method | FreeSurfer cross-sectional pipeline |
| Analysis software | FreeSurfer7.1.1. |
| Output measure  (e.g. absolute tissue volume in ml, tissue volume as a fraction of intracranial volume, percentage change in tissue volumes) | Mean cortical thickness using as weights the number of vertices of the regions involved |
| ***DTI*** | |
| Type  (e.g. whole brain, grey matter, white matter, spinal cord, normal-appearing grey matter or white matter) | Corticospinal tract, superior cerebellar peduncle, middle cerebellar peduncle and superior longitudinal fasciculus defined by the JHU combined atlas |
| Analysis method | TBSS |
| Analysis software | FMRIB's Diffusion Toolbox |
| Output measure | Fractional anisotropy and mean diffusivity |
| ***functional MRI*** | |
| Type  (e.g. whole brain, grey matter, white matter, spinal cord, normal-appearing grey matter or white matter) | Sensorimotor network (seeds: left and right primary motor cortices), default-mode network (seed: bilateral precuneus) and auditory network (seed: left primary auditory cortex) resting-state functional connectivity |
| Analysis method | Seed-based |
| Analysis software | CONN toolbox, REST software, REX toolbox |
| Output measure | Mean global diffusivity for each seed |
