# Supplementary material for: Contribution of structural and functional MRI in predicting response to motor training in multiple sclerosis
Source: Mult Scler. 2025 Dec 19;32(1):93–106. doi: 10.1177/13524585251398386 (PMC12756515; doi:10.1177/13524585251398386)
Supplement: sj-docx-2-msj-10.1177_13524585251398386 – Supplemental material for Contribution of structural and functional MRI in predicting response to motor training in multiple sclerosis [file sj-docx-2-msj-10.1177_13524585251398386.docx]

**Supplemental Methods**

**MRI acquisition**

The following brain MRI sequences were acquired from all subjects: (a) sagittal three-dimensional (3D) T1-weighted sequence (repetition time [TR]=7 ms; echo time [TE]=3.2 ms; inversion time [TI]=1000 ms; flip angle=8°; matrix size=256×256; field of view [FOV]=256×256 mm^2^; 204 contiguous sagittal slices, 1 mm thick); (b) variable flip angle 3D T2-weighted fluid-attenuated inversion recovery (FLAIR) turbo spin echo (TR=4800 ms; TE=270 ms; TI=1650 ms; matrix size=256×256; FOV=256×256 mm^2^; echo train length=167; 192 contiguous sagittal slices, 1 mm thick).

The following sequences were acquired for 53 multiple sclerosis (MS) patients and 31 healthy controls (HC): (c) pulsed-gradient spin echo (SE) echo planar imaging (EPI) (TE=58 ms, TR=8775 ms, acquisition matrix size=112x88, FOV=240x231 mm^2^, 55 contiguous, 2.3-mm thick axial slices, with SENSE [acceleration factor=2] and diffusion gradients applied in 35 non-collinear directions and b factor=900 s/mm^2^); (d) T2*‐weighted single‐shot EPI sequence for resting-state (RS) fMRI (TR=3000 ms, TE=35 ms, flip angle=85°, FOV=240x240 mm^2^, matrix=128×128, slice thickness=4 mm, 200 sets of 30 contiguous axial slices, parallel to the AC‐PC plane).

The following sequences were acquired for 35 MS patients and 39 HC: (e) axial pulsed-gradient spin echo single shot diffusion-weighted EPI (3 shells at b-value=700/1000/2855 s/mm^2^ along 6/30/60 non-collinear directions and 10 b=0 volumes, FOV=240×233 mm, pixel size=2.14×2.69 mm, 56 slices, 2.3 mm-thick, matrix=112×85, TR=5900 ms, TE=78 ms and three additional b=0 volumes with reversed polarity of gradients for distortion correction); (f) axial T2*-weighted single-shot EPI for RS fMRI (TR=1560 ms; TE=35 ms, flip angle=70°; multi-band factor=2, matrix size=96×96; FOV=240 x 240 mm^2^; 48 contiguous axial slices, 3 mm thick, number of volumes=320).

**MRI analysis**

Quantification of cortical thickness

Cortical thickness (Cth) was assessed on lesion-filled 3D T1-weighted images using the cross-sectional pipeline in FreeSurfer 7.1.1 analysis suite.^1, 2^ Several processing steps, including motion correction, skull stripping, Talairach transforms, atlas registration as well as spherical surface and cortical parcellations were performed. Output surfaces were inspected, and manual corrections were performed, if necessary (e.g., by removing skull structures from brain image or expanding white matter (WM) surface by applying special markers). Specific cortical areas were defined using the Human Brainnetome Atlas and the mean Cth of each area was extracted using as weights the number of vertices of the regions involved (http://atlas.brainnetome.org).^3^

DTI analysis

Preprocessing of diffusion-weighted imaging data included correction for off-resonance and eddy current induced distortions, as well as for subject movements, using the Eddy tool within the FSL library (FSL version 6.0.1, [www.fmrib.ox.ac.uk](http://www.fmrib.ox.ac.uk/)).^4^

Using the FMRIB's Diffusion Toolbox (FDT tool), the diffusion tensor was estimated in each voxel by linear regression using the shells at b≤1000 and fractional anisotropy (FA) and mean diffusivity (MD) maps were derived.^5, 6^ Individual FA images were non-linearly registered to the FMRIB58_FA atlas provided within FSL, and averaged. The resulting mean FA image was thinned to create a WM tract “skeleton” to include only WM voxels. Such WM “skeleton” was created by setting the FA threshold at a FA>0.2. Individual subjects’ FA values were projected onto this group skeleton by searching perpendicular from the skeleton for maximum FA values. Maximum FA values were chosen to restrict analysis to the center of WM tracts (where maximum FA values are found), rather than considering voxels at the edge of tracts, that may suffer from partial volume effects. The same procedure was applied for the analysis of mean diffusivity (MD) maps.

The middle cerebellar peduncle (MCP), superior cerebellar peduncle (SCP), corticospinal tract (CST) and superior longitudinal fasciculus (SLF), part of a combined atlas (JHU WM Labels-2 mm and JHU ICBM-Tracts-maxprob-thr25-2 mm), were overlaid to skeletonized FA maps from patients and HC to calculate average values in each region and globally.

fMRI analysis

fMRI data preprocessing was performed using CONN toolbox (https://web.conn-toolbox.org/).^7^ The RS fMRI scans were realigned to the mean of each session with a six-degree rigid-body using transformation to correct for head movements. After rigid registration of realigned images to the lesion-filled 3D T1-weighted scan, RS fMRI images were normalized to the MNI space using a nonlinear transformation. Smoothing was performed using a 3D 6-mm isotropic Gaussian filter. For denoising, the first five cerebro-spinal fluid and WM principal components were used as nuisance covariates in accordance with the anatomical component-based noise correction method. The six motion parameters and their first temporal derivates were regressed out of the data. Linear detrending and band-pass filtering (0.01-0.1 Hz) were performed to partially remove low-frequency drifts and high-frequency physiological noise.

Masks of the bilateral precuneus (obtained by merging left and right precuneus), as well as left and right Brodmann area 4 (primary motor cortices [M1]), left and right insulae and left Brodmann areas 41 and 42 (primary auditory cortices [A1]), were created by using the WFU PickAtlas toolbox (<http://fmri.wfubmc.edu/software/PickAtlas>).

A seed-based approach was then used to assess RS functional connectivity (FC) of these areas with the remaining brain regions.^8^ RS FC was assessed using REST software (https://www.nitrc.org/projects/rest/) by calculating the correlation coefficients between the time series extracted from the above-mentioned seeds and any other voxel in the brain. A Fisher’s z transformation was used to improve the Gaussianity of the obtained correlation coefficients.^7^ For each seed, mean global RS FC (mean Z-score of voxels within family-wise error [FWE] corrected mask of each network) was derived from SPM12 age- and sex-adjusted full factorial models and extracted using the REX toolbox (<https://www.nitrc.org/projects/rex>).

**Boruta feature selection and cross-validation analyses**

We applied the Boruta algorithm to select and rank baseline variables as predictors of training response.^9^ Baseline variables included demographic (age and sex), clinical (Expanded Disability Status Scale [EDSS], disease duration, phenotype, disease modifying treatment [DMT], 6-Minute Walking Test [6MWT], Timed 25-Foot Walk Test z-score [zT25FWT], peak oxygen consumption [VO_2_peak] and Modified Fatigue Impact Scale [MFIS]) and MRI (sequence; T2-hyperintense WM lesion volume [T2-LV]; normalized brain volume [NBV], normalized WM volume [NWMV], normalized cortical volume [NCV], normalized thalamic volume [NTV], anterior cerebellar motor area [ACMA] and posterior cerebellar motor area [PCMA] volumes; M1, precuneus, posterior cingulate gyrus [PCG], anterior cingulate gyrus [ACG], superior temporal gyrus [STG] and middle temporal gyrus [MTG] Cth; MCP, SCP, CST and SLF FA and MD; left and right M1, precuneus and left A1 mean global RS FC) measures. The analysis was performed separately within each training group and in the whole MS cohort, including training strategy as additional predictor. The algorithm builds Random Forest probability models (10,000 trees) and relies on importance scores (permutation importance, mean decrease in accuracy, internally computed on out-of-bag observations) to distinguish informative variables from those whose importance could arise by random chance. Specifically, Boruta iteratively (2,000 iterations) compares (using a binomial test with multiple-comparison adjustment) each real variable’s importance with the maximum importance achieved by shadow attributes, created by shuffling original ones. Features consistently exceeding the importance of the shadows are confirmed as relevant, while less informative variables are progressively discarded. This approach provides a robust, multivariable ranking of predictive features, capturing nonlinear effects and interactions, and is particularly suitable for datasets with many and correlated variables. We further assessed feature selection stability and the discriminative performance of the predictive models using a 5-fold, 50-times repeated cross-validation. Within each training fold, Boruta was applied to identify relevant predictors, and a Random Forest model was then fitted on the selected variables. Predictions for the corresponding held-out fold were pooled within each repetition, and cross-validated area under the curve (CV-AUC) values were computed. The percentage of times each predictor was selected across all folds and repetitions was recorded.

**Supplementary References**

1. Fischl B. FreeSurfer. *Neuroimage* 2012; 62: 774-781. 2012/01/18. DOI: 10.1016/j.neuroimage.2012.01.021.

2. Fischl B, Salat DH, Busa E, et al. Whole brain segmentation: automated labeling of neuroanatomical structures in the human brain. *Neuron* 2002; 33: 341-355. 2002/02/08. DOI: 10.1016/s0896-6273(02)00569-x.

3. Fan L, Li H, Zhuo J, et al. The Human Brainnetome Atlas: A New Brain Atlas Based on Connectional Architecture. *Cereb Cortex* 2016; 26: 3508-3526. 2016/05/28. DOI: 10.1093/cercor/bhw157.

4. Andersson JLR, Graham MS, Drobnjak I, et al. Towards a comprehensive framework for movement and distortion correction of diffusion MR images: Within volume movement. *Neuroimage* 2017; 152: 450-466. 20170308. DOI: 10.1016/j.neuroimage.2017.02.085.

5. Jones DK, Horsfield MA and Simmons A. Optimal strategies for measuring diffusion in anisotropic systems by magnetic resonance imaging. *Magn Reson Med* 1999; 42: 515-525.

6. Basser PJ, Mattiello J and LeBihan D. Estimation of the effective self-diffusion tensor from the NMR spin echo. *J Magn Reson B* 1994; 103: 247-254. 1994/03/01. DOI: 10.1006/jmrb.1994.1037.

7. Whitfield-Gabrieli S and Nieto-Castanon A. Conn: a functional connectivity toolbox for correlated and anticorrelated brain networks. *Brain Connect* 2012; 2: 125-141. 2012/05/31. DOI: 10.1089/brain.2012.0073.

8. Biswal BB, Mennes M, Zuo XN, et al. Toward discovery science of human brain function. *Proc Natl Acad Sci U S A* 2010; 107: 4734-4739. 20100222. DOI: 10.1073/pnas.0911855107.

9. Kursa MB and Rudnicki WR. Feature Selection with the Boruta Package. *J Stat Softw* 2010; 36: 1-13. DOI: DOI 10.18637/jss.v036.i11.
