# Supplementary material for: Contribution of structural and functional MRI in predicting response to motor training in multiple sclerosis
Source: Mult Scler. 2025 Dec 19;32(1):93–106. doi: 10.1177/13524585251398386 (PMC12756515; doi:10.1177/13524585251398386)
Supplement: sj-pdf-3-msj-10.1177_13524585251398386 – Supplemental material for Contribution of structural and functional MRI in predicting response to motor training in multiple sclerosis [file sj-pdf-3-msj-10.1177_13524585251398386.pdf]

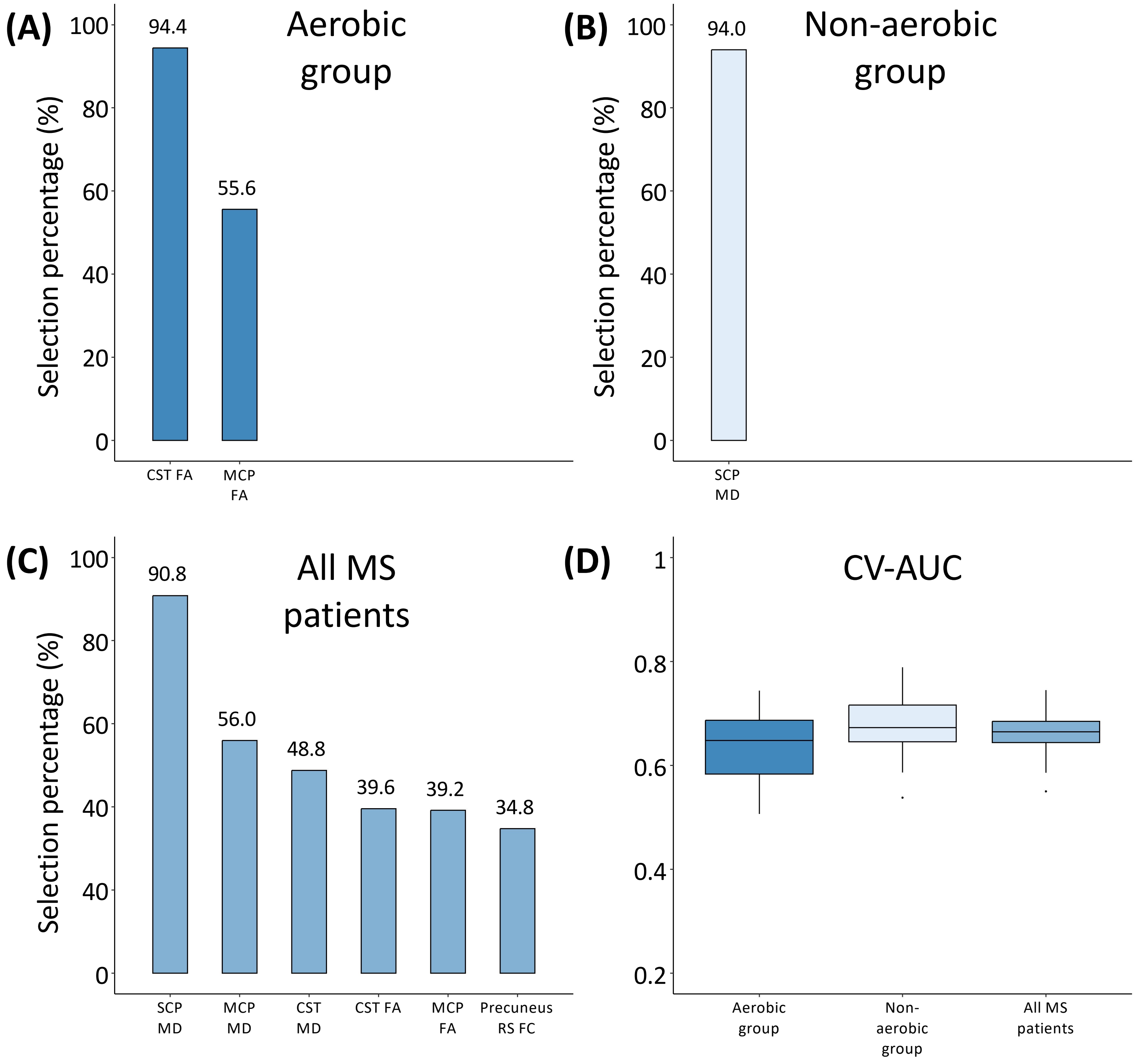

Supplementary Figure 1. Results of cross-validation analysis. Bar plots show predictors of response to training in (A) the aerobic group, (B) the non-aerobic group, and (C) all MS patients, identified by the Boruta algorithm in at least 30% of runs of the 50-times repeated 5-fold cross-validation. Panel (D) shows the distribution of CV-AUC values for each group.

Abbreviations: AUC=area under the curve; CST=corticospinal tract; CV=cross-validation; FA=fractional anisotropy; MCP=middle cerebellar peduncle; MD=mean diffusivity; MS=multiple sclerosis; SCP=superior cerebellar peduncle.
